# Impaired glycinergic transmission in hyperekplexia: a model of parasomnia overlap disorder

**DOI:** 10.1002/acn3.50866

**Published:** 2019-08-07

**Authors:** Régis Lopez, François Rivier, Jamel Chelly, Yves Dauvilliers

**Affiliations:** ^1^ Département de Neurologie, Unité des Troubles du Sommeil CHU Montpellier Montpellier France; ^2^ Inserm U1061 Montpellier France; ^3^ Department of Pediatric Neurology Neuromuscular Diseases Reference Center AOC CHU Montpellier France; ^4^ PhyMedExp University of Montpellier, INSERM, CNRS Montpellier France; ^5^ Service de Diagnostic Génétique Hôpital Civil de Strasbourg, Hôpitaux Universitaires de Strasbourg Strasbourg France; ^6^ IGBMC, INSERM, CNRS, Université de Strasbourg Strasbourg France

## Abstract

We report sleep phenotypes and polysomnographic findings in two siblings with a novel homozygous variant of the *GLRA1* gene causing hereditary hyperekplexia (HH). Both sisters had startles during wakefulness and sleep, sleep terrors, and one had symptoms of REM sleep behavior disorder (RBD). Frequent startles were found in NREM sleep associated with NREM parasomnias in deep sleep. In REM sleep, both had motor behaviors and increased phasic/tonic muscle activities confirming RBD. Clonazepam improved startles, motor behaviors, and muscle activities in REM sleep. Impaired glycinergic transmission in human HH could be involved in the pathophysiology of RBD and NREM parasomnias.

## Introduction

Hyperekplexia is a very rare neurological disorder characterized by exaggerated startle responses to unexpected stimuli, with a large spectrum of clinical severity (e.g. neonatal hypertonia, startle‐induced falls, intellectual disability).^1^ Hyperekplexia is caused by autosomal dominant or recessive modes of inheritance with more than 30 mutations reported in the alpha‐1 subunit of the glycine receptor gene, GLRA1,^2^ a ligand‐gated chloride channel that mediates postsynaptic inhibition mostly in the brainstem and spinal cord.

Glycinergic transmission is playing important roles in sensory signaling and motor functions, especially during Rapid Eye Movement (REM) sleep. Transgenic mice with a mutation in the *GLRA1* gene with deficiency in glycine and GABA‐A receptor function express most of the cardinal features of REM sleep behavior disorder (RBD), including REM motor behaviors, REM sleep phasic EMG motor activity but normal REM sleep atonia.^3^ These mice also presented myoclonic jerks during non‐REM (NREM) sleep and large sleep fragmentation. Another model of RBD was recently reported in rats after selective inactivation of the GABA/glycinergic transmission in the ventral medial medulla.^4^ The precise role of impaired GABA/glycine transmission in the pathophysiology of human RBD remains unknown.^5^

Only few studies were interested in assessing sleep characteristics in hyperekplexia.^6^, ^7^ Frequent startles were observed during NREM sleep, being almost absent in REM sleep. They were either spontaneous or triggered by a noise, light or unexpected somesthetic stimuli, with or without associated awakenings. In the first description of hyperekplexia, some patients had frequent comorbid somnambulism, somniloquy, and nightmares;^6^ however, the neurophysiological correlates of such parasomnias have not been studied.

Due to its unique pathophysiology, hereditary hyperekplexia is an interesting model to study the glycinergic pathway in motor control during sleep. We report here sleep phenotypes and video‐polysomnographic (vPSG) characteristics of two siblings affected with hyperekplexia.

## Case Series

Two sisters (patient #1: 25 y.o and #2: 17 y.o.), born of consanguineous parents, with autosomal recessive hyperekplexia were admitted to the sleep laboratory for evaluation. Genetic testing of the two cases revealed a novel homozygous variant c.57‐1G>T, likely to cause loss of function of the *GLRA1* gene (NM_001146040.1), that was not reported in any of the public databases. Both parents were also analyzed and found heterozygous for this variant.

Both patients had a history of exaggerated startle reflexes and falls in response to loud noises, with continuous treatment with clonazepam (1–2 mg per day) since early childhood with moderate response. They both had a history of delay in speech acquisition and mild intellectual disability, and generalized anxiety for patient #1. Brain imaging revealed an asymptomatic type 1 Chiari malformation in patient #1, while brain MRI was unremarkable in patient #2.

Since early childhood, both sisters reported brief and repeated sudden awakenings associated with startles during nighttime, and frequent sleep terrors characterized by sudden awakening associated with behavioral manifestations of intense fear, frightening screams and amnesia in the first part of the night. These abnormal behaviors persisted almost daily despite clonazepam intake. Patient #1 also had weekly episodes of complex behaviors such as gesturing, screaming, laughing associated with dream content since childhood, mostly in the second part of the night suggesting RBD. These abnormal behaviors are observed by the parents and sisters as they continue to share the same bedroom.

Both sisters had neither daytime sleepiness nor symptoms of sleep breathing disorder. Patient #1 complained on sleep onset insomnia since 5 years of age. No sleep problems were found among family members.

Two consecutive vPSGs were performed for each sister in the laboratory (first night with clonazepam; second night without). Sleep stages, microarousals, periodic limb movements, and respiratory events were scored manually according to standard criteria. A particular attention was paid to motor behaviors, tonic and phasic chin muscle activities during REM sleep, and N3 sleep fragmentation.^8^, ^9^ Results were compared to normative values obtained in 15 healthy controls (10 males, median age 24 years, range 19–30 years).

**Figure 1:**
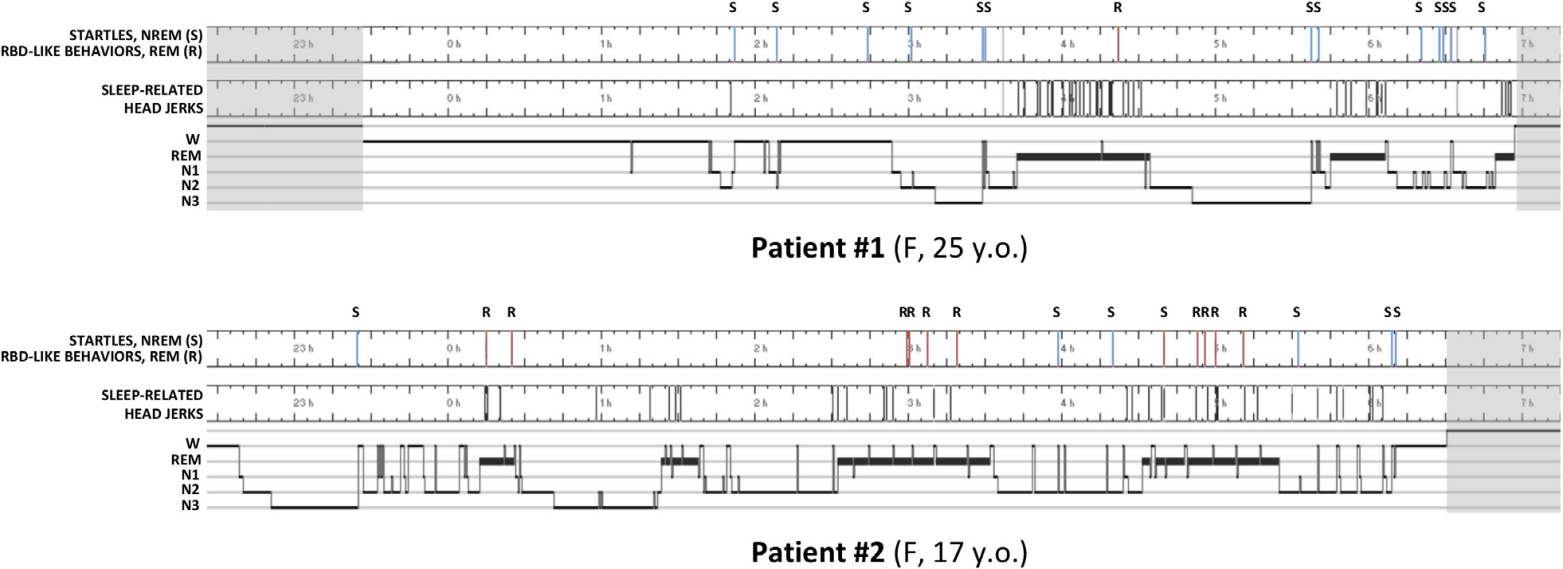
Polysomnography and distribution of motor behaviors across the night in two siblings with hereditary hyperekplexia.

**Figure 2:**
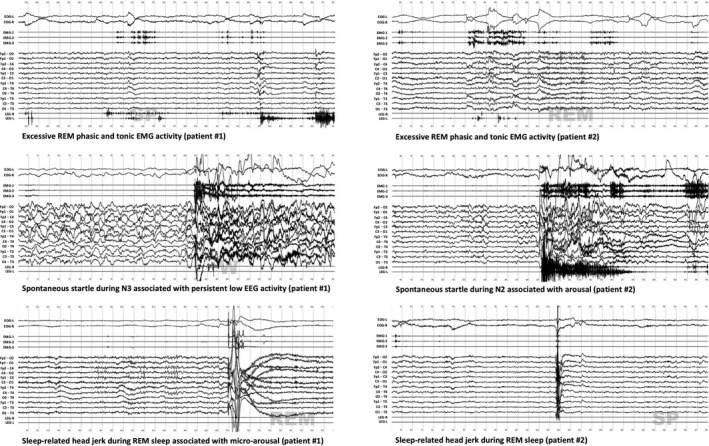
Polysomnographic characteristics of REM and NREM motor activities in two siblings with hereditary hyperekplexia.

Patient #1 had reduced sleep efficiency with prolonged sleep onset latency while sleep architecture and continuity were preserved in Patient #2 (Table 1, Fig. 1). However, a high percentage of REM sleep was observed in both patients with or without clonazepam intake. Respiratory events and periodic limb movements were in the normal range but slightly increased after clonazepam intake. On the untreated nights, frequent spontaneous startles were observed in both patients in NREM sleep only, associated with microarousals in N1 and N2 sleep, and with some sleep terrors with concomitant slow postarousal EEG activity in N3 sleep. No similar startles were found in REM sleep; however simple motor behaviors involving the face and upper limbs, and frequent sleep‐related head jerks (SRHJ), characterized by sudden flexion or version of the head were found in REM sleep in both sisters.^10^, ^11^ Phasic and tonic chin mentalis EMG activities were also increased in REM sleep, being above the pathological cut off in both patients for the phasic (>15%) and for patient #1 for the tonic activity (>30%) (Fig. 2). Clonazepam resulted in a dramatic decrease of startles in NREM sleep and decrease in motor behaviors with improvement in tonic and phasic EMG activities in REM sleep.

**Table 1:** Video‐polysomnographic characteristics of two siblings with hereditary hyperekplexia in untreated and treated conditions and healthy controls.

	Patient #1 Female 25 y.o.		Patient #2 Female 17 y.o.		Controls *N* = 15	
	Untreated	Clonazepam 2 mg	Untreated	Clonazepam 1 mg	Mean (SD)	Median [min‐max]
Polysomnographic characteristics						
Total sleep time (min)	253	399	415	380	405.3 (53.8)	429 [289–472]
Sleep efficiency (%)	55.8	87.4	85.1	73.3	85.1 (9.9)	87 [62–96]
Sleep latency (min)	108	46	16	23	24.4 (20.9)	17 [4–84]
N1 (%)	12.8	3.1	4.6	3.4	3.6 (1.8)	3.0 [1.0–6.0]
N2 (%)	29.8	30.7	47.1	46.1	55.5 (7.0)	56.0 [43.0–49.0]
N3 (%)	25.7	21.9	17.6	20.7	23.7 (6.2)	24.0 [12.0–36.0]
REM (%)	31.6	44.3	30.8	27.1	17.1 (4.4)	17.0 [8.0–26.0]
PLM index (/h)	7.8	10.2	5.8	9.6	1.1 (2.4)	0.0 [0.0–8.7]
AHI (/h)	–	1.1	–	7.1	2.1 (2.4)	1.5 [0.0–9.0]
Microarousal index (/h)	16.6	14.9	14.0	9.5	11.2 (5.9)	9.4 [3.2–23.9]
Sleep‐related motor activities and behaviors						
Startles, NREM (*n*)	13	1	6	1	0	
Startles, N1 (*n*)	3	0	1	0		
Startles, N2 (*n*)	8	0	4	0		
Startles, N3/ with ST episode (*n*)	2/2	1/0	1/1	1/0		
SWSFI (/h)	5.5	2.7	2.5	3.0	4.2 (2.5)	4.5 [1.3–7.8]
Slow/Mixed Index (/h)	4.6	2.7	2.5	3.0	2.2 (2.0)	1.3 [0.0–5.7]
Simple motor behaviors, REM (*n*)	1	2	11	4	0	
Phasic EMG mentalis, REM (%)	40.6	11.2	21.0	17.3	3.8 (2.9)	2.8 [0.0–10.4]
Tonic EMG mentalis, REM (%)	40.0	3.7	19.5	10.7	0.7 (1.3)	0.0 [0.0–4.6]
SRHJ index, REM (/h)	27.8	28.2	11.7	8.7	1.4 (2.1)*	0.0 [0.0–7.0]*

Abbreviations: PLM, Periodic Limb movements; AHI, Apnea Hypopnea Index; REM, Rapid Eye Movement Sleep; NREM, Non Rapid Eye Movement Sleep; EMG, electromyogram; SRHJ, Sleep‐Related Head Jerks.

* Six of 15 controls had at least one SRHJ.

## Discussion

We documented abnormal behaviors and motor activities during NREM and REM sleep, with objective improvement with clonazepam, in two siblings affected with hereditary hyperekplexia caused by a novel splice mutation affecting the consensus splice acceptor site of intron 2 of the *GLRA1* gene. This *GLRA1* variant is an intronic single nucleotide substitution that affects the canonical splice acceptor site of exon 2 and all queried bioinformatics algorithms predicted an abolition (estimated at 100%) of the concerned acceptor splice site of exon 2. This in turn would lead to exon 2 skipping and a transcript lacking exon 2 with a disrupted reading frame and a premature termination codon (PTC) in exon 3 (p.Ser19Argfs*20). Although further investigations are required to assess the consequences of the mutations on mRNA stability and protein expression, unnatural splice sites introducing a PTC into the proximal part of mRNA often lead to nonsense‐mediated decay of the transcript leading to a loss of function of the gene.

Both sisters exhibited frequent startles during NREM sleep, a phenotype previously described in early reports of hyperekplexia without identification of *GLRA1* gene mutations.^6^, ^7^ During N3 sleep, spontaneous startles were associated with sleep terrors and persistent slow postarousal EEG activity, a pattern typically observed in disorders of arousal (DOA),^12^ these parasomnias being previously reported in hyperekplexia patients. Both sisters were just above the slow/mixed arousal index but below the threshold of slow wave sleep fragmentation index for DOA.^9^ Other motor behaviors involving the face and the upper limbs were found in REM sleep, together with increased phasic and tonic chin mentalis EMG activities in both patients. The association of such motor behaviors in REM sleep and a complaint of dream‐enactment behaviors meet the RBD diagnostic criteria for patient #1. The occurrence of RBD has been previously described in two old patients with symptomatic hyperekplexia.^13^, ^14^ In patient #1, we cannot formally exclude the role of the type 1 Chiari malformation in generating RBD.^5^ We finally observed frequent SRHJ in REM sleep, being higher than observed in our control group, and normative values.^10^

Recent studies in mice and rats demonstrated the major role of inhibitory GABA/glycinergic synapses in motor pathways of the brainstem. Genetic inactivation in GABA/glycine neurotransmission in the ventromedial medulla disrupts muscle atonia during REM sleep and triggers abnormal behaviors without any effect during waking and slow‐wave sleep.^4^ Transgenic mice with deficient glycine and GABA‐A receptor function exhibited increased motor activity during NREM and REM sleep, NREM myoclonic jerks, sleep fragmentation, and increased phasic but normal tonic motor activities in REM sleep.^3^ All these findings were found in our cases with hereditary hyperekplexia. A global motor dyscontrol has also been reported in patients with idiopathic RBD, with increased periodic and nonperiodic limb movements in NREM and REM sleep, involving both legs and arms and occurring throughout the entire sleep cycle;^15^ however NREM parasomnias such as sleep terror and sleepwalking were rarely seen in patients with idiopathic RBD, who are often elderly.^5^ In the same way, in patients with DOA, who are often young subjects, behavioral episodes suggestive of RBD were rarely seen.^12^ However, two previous studies reported a higher amount of phasic with or without increased tonic EMG activity in REM sleep in patients with DOA that warrants further confirmation.^16^, ^17^ Startles and both NREM and REM parasomnias were improved after clonazepam treatment. Interestingly, clonazepam also rescued this complex phenotype in transgenic mice, certainly in mediating glycinergic/GABAergic inhibitory neurotransmission in the brainstem.^3^

In conclusion, we reported abnormal behaviors and motor activities during NREM and REM sleep in two siblings affected with hereditary hyperekplexia caused by a mutation in the *GLRA1* gene, with the majority of motor defects being successfully treated by clonazepam. The present cases emphasized the need to better investigate sleep disorders in hereditary hyperekplexia, being a cause of overlap parasomnia disorder (e.g RBD and DOA) especially in young subjects.^18^ Our findings suggest that impaired glycinergic transmission may be involved in the pathophysiology of RBD and DOA. Pharmacological glycine receptor potentiation warrants investigations in patients with parasomnia overlap disorder and may represent a promising therapeutic approach.

## Ethical Publication Statement

We confirm that we have read the Journal's position on issues involved in ethical publication and affirm that this report is consistent with those guidelines.

## Conflict of Interest

RL received funds for seminars and travel by UCB Pharma, HAC pharma and Shire. JC reports no disclosures. FR received funds for travel by Sarepta international France. YD received funds for seminars, board engagements, and travel by UCB Pharma, Jazz, Theranexus, Flamel, and Bioprojet.

## Author Contribution

RL Designed and executed the study, and wrote the manuscript. FR reviewed the manuscript. JC performed genetic analyses and reviewed the manuscript. YD designed the study and reviewed the manuscript.
